# Involvement of the CXCR7/CXCR4/CXCL12 Axis in the Malignant Progression of Human Neuroblastoma

**DOI:** 10.1371/journal.pone.0043665

**Published:** 2012-08-20

**Authors:** Julie Liberman, Hervé Sartelet, Marjorie Flahaut, Annick Mühlethaler-Mottet, Aurélie Coulon, Carine Nyalendo, Gilles Vassal, Jean-Marc Joseph, Nicole Gross

**Affiliations:** 1 Pediatric Oncology Research Unit, Department of Pediatrics, Lausanne University Hospital (CHUV), University of Lausanne, Lausanne, Switzerland; 2 Department of Pathology, Sainte Justine University Hospital, Montreal, Quebec, Canada; 3 Department of Molecular Medicine, Sainte Justine University Hospital, Montreal, Quebec, Canada; 4 UPRES UE 3535, Institut Gustave Roussy, Villejuif, France; 5 Department of Pediatric Oncology, Institut Gustave Roussy, Villejuif, France; 6 Division of Pediatric Surgery, Department of Pediatrics, Lausanne University Hospital, University of Lausanne, Lausanne, Switzerland; University of Florida, United States of America

## Abstract

Neuroblastoma (NB) is a typical childhood and heterogeneous neoplasm for which efficient targeted therapies for high-risk tumors are not yet identified. The chemokine CXCL12, and its receptors CXCR4 and CXCR7 have been involved in tumor progression and dissemination. While CXCR4 expression is associated to undifferentiated tumors and poor prognosis, the role of CXCR7, the recently identified second CXCL12 receptor, has not yet been elucidated in NB. In this report, CXCR7 and CXCL12 expressions were evaluated using a tissue micro-array including 156 primary and 56 metastatic NB tissues. CXCL12 was found to be highly associated to NB vascular and stromal structures. In contrast to CXCR4, CXCR7 expression was low in undifferentiated tumors, while its expression was stronger in matured tissues and specifically associated to differentiated neural tumor cells. As determined by RT-PCR, *CXCR7* expression was mainly detected in N-and S-type NB cell lines, and was slightly induced upon NB cell differentiation *in vitro*. The relative roles of the two CXCL12 receptors were further assessed by overexpressing CXCR7 or CXCR4 receptor alone, or in combination, in the IGR-NB8 and the SH-SY5Y NB cell lines. *In vitro* functional analyses indicated that, in response to their common ligand, both receptors induced activation of ERK1/2 cascade, but not Akt pathway. CXCR7 strongly reduced *in vitro* growth, in contrast to CXCR4, and impaired CXCR4/CXCL12-mediated chemotaxis. Subcutaneous implantation of CXCR7-expressing NB cells showed that CXCR7 also significantly reduced *in vivo* growth. Moreover, CXCR7 affected CXCR4-mediated orthotopic growth in a CXCL12-producing environment. In such model, CXCR7, in association with CXCR4, did not induce NB cell metastatic dissemination. In conclusion, the CXCR7 and CXCR4 receptors revealed specific expression patterns and distinct functional roles in NB. Our data suggest that CXCR7 elicits anti-tumorigenic functions, and may act as a regulator of CXCR4/CXCL12-mediated signaling in NB.

## Introduction

Neuroblastoma (NB) is a typical pediatric neoplasm derived from embryonic neural crest cells. The tumor recapitulates characteristics of its originating cells, with an extensive heterogeneity, pluripotential differentiation and migratory abilities. The disease displays a remarkable clinical diversity, ranging from spontaneous regression to fatal progression and dissemination to privileged sites, such as bone-marrow and liver [1], [2].

Chemokines and their receptors have been originally described as essential mediators of leukocyte directional migration, particularly during infection and inflammation, and have further emerged as crucial players in all stages of tumor development [3], [4], [5], [6]. The binding of chemokines to their cognate receptors elicits typical cellular responses, such as directional migration, through activation of classical MAP-kinase or PI3-kinase/PKB signaling cascades [7]. Both tumor and stromal cells express a large pattern of chemokine/chemokine receptor axes, which may represent major paracrine/autocrine complex players within the tumor and its microenvironment [8].

CXCR4 is the most frequently expressed chemokine receptor on tumor cells [9]. In addition to its critical role in tumor cell growth, survival and angiogenesis in multiple cancers, the CXCR4/CXCL12 pair has been shown to mediate homing and metastatic secondary growth in CXCL12-producing organs, such as liver and bone marrow [10], [11], [12]. However, the relative contribution of the CXCR4/CXCL12 axis in organ-specific dissemination or/and tumor growth has been strongly debated [13], [14], [15]. In particular, we have previously shown that CXCR4 mostly promoted NB primary tumor and secondary growth, without influencing organ-specific dissemination of malignant NB cells [16].

CXCR4 has long been considered as the only mediator of CXCL12-induced biological effects. CXCR7, formerly called RDC1, has been recently identified as an alternate receptor for CXCL12 [17], [18]. This new chemokine receptor has been demonstrated to bind with high affinity to CXCL12 and with low affinity to a second chemokine, interferon-inducible T cell chemoattractant (I-TAC; also known as CXCL11). However, despite its chemokine receptor phylogenic relation, growing evidence has suggested that CXCR7 does not mediate typical chemokine responses such as G protein-coupled receptor-mediated calcium mobilization [17], [18], [19], [20]. Although coupling of the CXCR7 receptor with G proteins is still under debate [20], [21], the possibility that the receptor is able to induce signal transduction is nevertheless suggested by reports demonstrating MAPK and Akt pathway activation upon CXCR7-expressing cell exposure to CXCL12 [22], [23], [24]. In humans, CXCR7 is expressed in embryonic neuronal and heart tissue, in some hematopoietic cells, and activated endothelium [17], [18], [25], [26], [27], [28], [29]. Elevated levels of CXCR7 have been detected in several tumors, and particularly in the endothelial cell-associated vasculature [10], [24], [30]. Moreover, CXCR7 expression was shown to promote growth and metastasis of various tumor models *in vivo*, suggesting a role for CXCR7 in regulating immunity, angiogenesis, and organ-specific metastasis [10], [30], [31]. The identification of CXCR7 has added new perspectives for the implication of the CXCR4/CXL12 pair in tumor biology [32], [33]. However, CXCR7 specific implication in NB dissemination, and its contribution to CXCL12/CXCR4–mediated NB signaling are still not fully elucidated.

In this study, we investigated the expression of CXCR7 and CXCL12 in a large panel of NB tissues. Our data revealed a generally low CXCR7 expression in primary NBs of all stages, which was however specifically enhanced in neural-associated compartment of differentiated and matured tumors. *In vitro* and *in vivo* studies showed that CXCR7 elicited anti-tumorigenic properties, particularly in presence of CXCR4. Indeed, we suggested that CXCR7 was sufficient to act as a regulator of specific CXCR4/CXCL12-mediated NB growth and migration. Altogether our data pointed to the existence of a putative cross-talk between the two CXCL12 receptors in NB cell lines, and suggested the implication of the global CXCR7/CXCR4/CXCL12 axis in the regulation of NB progression.

## Materials and Methods

### Ethics Statement

Our study using patient tissues was approved by the ethical research review board of the State University Hospital of Lausanne (CHUV). Patient tissue collection and analyses were performed after patients’ written informed consent. Animal experimentation protocols (authorization number: 1564.1/5) were approved by the state veterinary services.

### Cell Lines

All NB well-characterized cell lines [34], the MCF-7 breast cancer cell line [17], the PC-3 prostate cancer cell line [35], and the SW480 colon cancer cell line [36] were cultured in Dubelcco’s modified Eagle’s medium (D-MEM) (Gibco, Paisley, UK) supplemented with 1% penicillin/streptomycin (Gibco) and 10% heat inactivated Foetal Calf Serum (FCS) (Sigma-Aldrich, St Louis, MO, USA).

### Tissue-microarray (TMA)

The TMA was composed of tumor samples from 156 patients, diagnosed with NB between July 1988 and April 2002, treated and followed in four clinical centers: Bicêtre hospital and Gustave Roussy Institute (Villejuif, France), the American Hospital (Reims, France), CHU Sainte Justine (Montréal, Canada), and Shiga University hospital (Otsu, Japan). Four tissue cylinders (0.6 mm diameter) per sample were obtained and transferred into a recipient paraffin block using a manual tissue arrayer (Alphelys, Plaisir, France). Five µm sections of TMA blocks were deparaffinated in xylol bath for 10 min, and rehydrated by successive transfers in alcohol baths with decreasing concentration, and finally in H_2_0. Then, sections were washed for 5 min in 3% H_2_O_2_ to inhibit endogenous peroxydase. TMA sections were incubated overnight at 4°C with mouse anti-human CXCL12 (clone 79018, R&D systems, Minneapolis, MN, USA) and mouse anti-human CXCR7 (clone 9C4, kind gift from Dr. M. Thelen, IRB, Bellinzona, Switzerland) antibodies diluted in Dako REAL™ antibody diluent (Dako, Glostrup, Denmark). Incubation with secondary antibody was performed using EnVision™ HRP-antibodies (Dako) for 30 min, followed by treatment with 100 µl DAB (Dako) at 1/50 dilution for 8 min. Slides were then incubated in hematoxylin bath for 10 s, and then dehydrated in baths with increasing alcohol concentration, and finally in xylol. Washes between each step were done in TBS pH 7.6. Slides were mounted using Eukitt Mounting Medium (EMS, Hatfield, PA, USA). Immunostaining scores (0–4) were established for each stained tissue by semi-quantitative optical analysis by two independent investigators blinded for clinical data. The percentage of positive cells in each sample was scored as follows: 0, all cells negative; 1+, up to 25% of cells were positive; 2+, 26% to 50%; 3+, 51% to 75%; 4+, more than 75%.

### RT-PCR

Total RNA, extracted from cell lines using the RNeasy Mini kit (Qiagen, Hilden, Germany), was reverse-transcribed using PrimeScript™ RT reagent Kit, according to the manufacturer’s instructions (TAKARA Bio Inc., Shiga, Japan). One µl of cDNA was added to 5 U/µl GoTaq® Hot Start Polymerase (Promega, Madison, MI, USA), specific buffer, 0.2 mM dNTPs and 1 µM human-specific primer pairs. The PCR reaction consisted of 2 min at 95°C, followed by 30 cycles of 30 s at 95°C, 30 s at 60°C, and 30 s at 72°C, with a final step of 5 min at 72°C. *CXCR4* and *CXCR7* expression levels were compared to those of the *GAPDH* housekeeping gene. PCR products were analyzed on 2% agarose gels. Real-time semi-quantitative RT-PCR was performed using the ABI PRISM 7900 HT real-time PCR system (Applied Biosystem) with SYBR Green© detection mix (Qiagen). Expression levels of *CXCR4* and *CXCR7* transcripts were calculated relatively to the level of the housekeeping gene *HPRTI* using the ΔΔC_t_ method. PCR program corresponded to: 2 min at 50°C, 5 min at 95°C, 40 cycles of three repeated steps of amplification (10 s at 95°C, 30 s at 60°C, 15 s at 95°C), and 15 s at 65°C. Human-specific pairs of primers: *CXCR7*∶5′-TGGGCTTTGCCGTTCCCTTC-3′ and 5′-TCTTCCGGCTGCTGTGCTTC-3′, *CXCR4*∶5′-TATCTGTGACCGCTT-CTACC-3′ and 5′-GCAGGACAGGATGACAATA-C-3′, *GAPDH:* 5′-AGATCATCAGCAATGCCTCC-3′ and 5′-GTGGCAGTGATGGCAT-GGAC-3′, *HPRT1∶*5′-TGACACTGGCAAAACAATGCA-3′ and 5′-GGTCCTTTTCACC-AGCAAGCT-3′.

### Plasmids

The complete coding sequence of *CXCR7* (1.089 kb) was amplified by PCR from a pcDNA3 plasmid containing human *CXCR7* (kindly provided by Dr. M. Thelen, Bellinzona) using 5′ and 3′ primers containing XhoI and EcoRI sites as follows: sense: 5′-GCGCCTCGAGATGGATCTGCATCTCTTCGACTAC-T-3′; antisense: 5′-GCGCG-AATTCTCATTTGGTGCTC-TGCTCCA-3′. The amplified cDNA was subcloned into the pMigr vector (kind gift from F. Louache, Institut Gustave Roussy, Villejuif, France) containing IRES-EGFP sequence. The pMigr plasmid containing complete coding region of *CXCR4* (1.1 kb) was already used and described elsewhere [16], [37]. Plasmids containing *CXCR7* and *CXCR4* cDNAs were sequenced for their integrity. The pMigr-EGFP vectors encoding for EGFP with or without *CXCR4* or *CXCR7* gene was inserted by retroviral-mediated infection into NB cells, as previously described [37].

### Flow Cytometry

Transduced GFP-expressing cells were sorted by FACS AriaI™ cell sorter (BD Biosciences, San Jose, CA, USA) to control transfection efficiency.

Single cells were stained with PE-labeled mouse anti-CXCR4 (clone 12G5, BD Biosciences), and mouse anti-CXCR7 (clone 9C4), as previously described [16], [18]. Alexa Fluor® 647-labeled goat anti-mouse secondary antibody (Invitrogen, Carlsbad, CA, USA) was used for the detection of CXCR7. Ten thousand events were analyzed by FACScan (BD Biosciences).

### Immunofluorescence

Hundred thousand cells were plated in Lab-Tek^R^ Chamber Slide™ System (Nunc, Ny,USA), 48 h before analyses. Cells were washed with PBS, fixed in 4% paraformaldehyde (PFA) (Fluka, Buchs, Switzerland) for 10 min at room temperature, and then permeabilized with SAP buffer (0.1% saponin (Sigma)−0.05%NaN_3_ in PBS) for 15 min [22]. Cells were blocked in SAP buffer supplemented with 10% goat serum (Sigma), and then incubated with anti-CXCR7 (10 µg/mL clone 9C4, and clone 11G8 from R&D systems) or anti-β_3_ tubulin (1∶1000, clone 2G10, Sigma) in SAP buffer containing 1.5% goat serum. Cells were next incubated with appropriate Cy3-conjugated secondary antibody (Jackson ImmunoResearch Laboratories, West Grove, PA, USA). DAPI (Sigma) was added for nuclear staining, and slides were mounted using DAKO® Fluorescent mounting medium (Dako). Imaging was performed using a camera DFC345 FX (Leica Microsystems Schweiz AG, Switzerland) and analyzed with the Leica Application Suite (LAS) software.

### Differentiation Assay

*In vitro* neuronal and glial/shwannian differentiation assays were performed by treating NB cells with all-trans retinoic acid (RA) (Sigma) and bromodeoxyuridine (BrdU), respectively, as previously described [38], [39], [40]. RA was dissolved in DMSO to a concentration of 3.5 mg/ml and stored in light protected vials at −20°C. Aliquots of stock solution were freshly thawed for each experiment and diluted in DMEM, 10% FCS. NB cells were plated 24 h before treatment with either 10 µM RA or BrdU. Medium was renewed every three days.

### ERK1/2 and Akt Phosphorylation

Following overnight serum starvation, cells were either unstimulated or stimulated with 100 ng/mL human recombinant CXCL12 or CXCL11 (PeproTech, Rocky Hill, NJ, USA) for indicated time, or pre-treated with 1 µM of the specific CXCR4 blocker 4F-benzoyl TN14003 (kind gift of N.Fujii, Kyoto, Japan) prior to ligand stimulation. Cells were lysed in sample buffer (250 mM Tris-HCl at pH 6.8, 10% SDS, 40% Glycerol, 16% β-mercaptoethanol, 0.04% Bromo-phenol-blue), and protein lysates were loaded on 10% SDS-PAGE. Gels were transferred to Immobilon-P membranes (Millipore, Volketswil, Switzerland). Membranes were blocked in TBS-Tween 0.01% containing 2% ECL Advance™ Blocking Agent (Amersham™ ECL Advance™ Western Blotting Detection Kit, GE Healthcare, Buckinghamshire, UK), and blotted with specific primary antibodies: rabbit anti-phospho-p44/42 MAPK (thr202/Tyr204), rabbit anti-phospho-Akt (Ser 473), rabbit anti-p44/42 MAPK, rabbit anti-Akt (all from Cell Signaling, Danvers, MA, USA). Blots were then incubated with the appropriate HRP-conjugated secondary antibody (Dako). ECL detection kit (GE Healthcare) was used for detection.

### Soft Agar Assay

Anchorage-independent colony formation assay, modified from a previous protocol [41], was performed using double-layer soft agar in 6-well plates (Corning) with a top layer of 0.175% agar (Difco™ Agar Noble, BD Biosciences) and a bottom layer of 0.35% agar. Briefly, 5×10^3^ NB cells were suspended in 0.175% agar diluted in DMEM/10% FCS, and laid on the top of the supporting agar layer. When stipulated, 100 µL fresh medium supplemented with 100 ng/mL CXCL12 was added weekly. Colonies were allowed to form at 37°C for at least two weeks. Colony cell viability was assessed using the MTS/PMS cell proliferation kit (Promega), and viable colonies were counted using light microscopy (Leica Laborluc D).

### Chemotaxis Assay

Cell migration was measured using Transwell Costar® cell culture chambers with polycarbonate filters of 8 µm porosity (BD Biosciences), as previously described [16]. 2×10^5^ cells suspended in DMEM/2% FCS were seeded in the upper compartment of the chamber system. The lower compartment was filled with DMEM/2% FCS supplemented or not with 100 ng/ml CXCL12 (PrepoTech). The cells were allowed to settle down for 4 h. After washing with PBS, membranes were fixed for 10 min in 4% PFA (Fluka) in PBS. Membranes were stained with haematoxylin (Polysciences, warrington, PA, USA). Non-migrated cells were carefully scraped from the upper side of the filter, and migrated cells on the lower side were counted by light microscopy.

### In vivo Studies

All animal experiments were carried out with Swiss athymic nude mice (Balb/C nu/nu). For heterotypic assays, groups of three mice were subcutaneously injected in the flank with 2×10^5^ cells suspended in 200 µl mix (1∶1) of DMEM and BD Matrigel™ Basement Membrane Matrix (BD Biosciences). The grafted animals were then weekly monitored with calipers for tumor growth assessment. The tumor volume was calculated using the formula (length×width^2^)/2. For orthotopic assays, seven animals per cell line were engrafted with NB cells directly in the left adrenal gland, as previously described [16], [37]. Briefly, 5×10^5^ cells in 15 µl DMEM were injected in the adrenal gland using a 22G needle connected to a Hamilton syringe. Tumor take and growth were followed by ultrasound imaging every 10 days, at the Lausanne Cardiovascular Assessment Facilities. Macroscopic metastases were assessed by gross examination.

### CXCL12 ELISA

Cultured NB cells were harvested and suspended in RIPA lysis buffer (25 mM HEPES pH 7.4, 150 mM NaCl, 10% glycérol, 1.5 mM MgCl2, 1% Triton X-100, 1% sodium deoxycholate, 0.1% SDS, 100 mM NaF), supplemented with a protease inhibitor cocktail (Complete mini, EDTA-free, Roche, Mannheim, Germany). Snap frozen tumors and mouse tissues were cut in small pieces and suspended in the above described lysis buffer. Samples were sonicated for 30 s, followed by a centrifugation step for 15 min at 20’000 g. Total protein amount was quantified using the Bradford method (Biorad Laboratories, Richmond, CA, USA). CXCL12 expression levels were quantified using a CXCL12 ELISA kit (R&D Systems) according to the manufacturer’s guide.

### Statistical Analyses

Statistical analyses were performed using GraphPadPrism 5.0 (GraphPad Software Inc., San Diego, CA, USA). *p<0.05 represented significance; **p≤0.01 and ***p≤0.001 were interpreted to be highly significant.

## Results

### Expression of CXCR7 and CXCL12 in NB Tissues

A NB TMA including a panel of 156 primary NB tumors, 56 metastatic and 65 control normal tissues, such as adrenal glands (AG) and sympathetic ganglia (SG), was screened for CXCR7 and CXCL12 expression. Patient clinical data and associated tumors are detailed in Table 1. The expression of CXCR7 or CXCL12 was semi-quantitatively assessed as an immunostaining score (0–4) in three distinct cell populations in each tissue: the neural, endothelial and stromal compartments. Neuroblasts and tumor ganglion cells were included in the neural compartment of tumors, while adrenal medulla and normal ganglion cells represented the neural part of AG and SG, respectively. Fibroblasts in tumors and AG, and Schwann cells in tumors and SG were attributed to the stroma.

**Table 1:** TMA: clinical characteristics.

Patient at diagnosis	N = 156
**Age (mo)**	
Median (range)	26 (0–151)
<12 mo, n (%)	78 (50)
≥12 mo, n (%)	78 (50)
**Follow-up (mo)**	
Median (range)	101 (1–243)
**Survival**	
Alive at time of last follow-up, n (%)	117 (75)
**INSS stage**	
1, n (%)	31 (20)
2, n (%)	19 (12)
3, n (%)	32 (21)
4, n (%)	58 (37)
4S, n (%)	16 (10)
**COG Risk Classification**	
Low, n (%)	54 (35)
Intermediate, n (%)	44 (28)
High, n (%)	58 (37)
**Neuroblastoma type**	
Standard, n (%)	101 (65)
Mass screening, n (%)	55 (35)
**Sample type**	
Primary tumor, n	156
Metastasis, n	56
*Lymph node, n (%)*	*48 (86)*
*Liver, n (%)*	*6 (10)*
*Skin, n (%)*	*2 (4)*
Control normal tissue, n	65
*Adrenal Gland, n (%)*	*50 (77)*
*Sympathetic ganglion, n (%)*	*15 (23)*
**Differentiation Stage**	
UnNB, n (%)	36 (23)
GGNB, n (%)	20 (12)
GGN, n (%)	6 (3)

mo: month.

n: number of cases.

INSS: International Neuroblastoma Staging System.

COG: Children Oncology Group.

NB: neuroblastoma.

UnNB: undifferentiated NB.

GGNB: ganglioneuroblastoma.

GGN: ganglioneuroma.

#### CXCR7 is preferentially expressed by mature neural cells in differentiated and matured tumors

**Figure 1:**
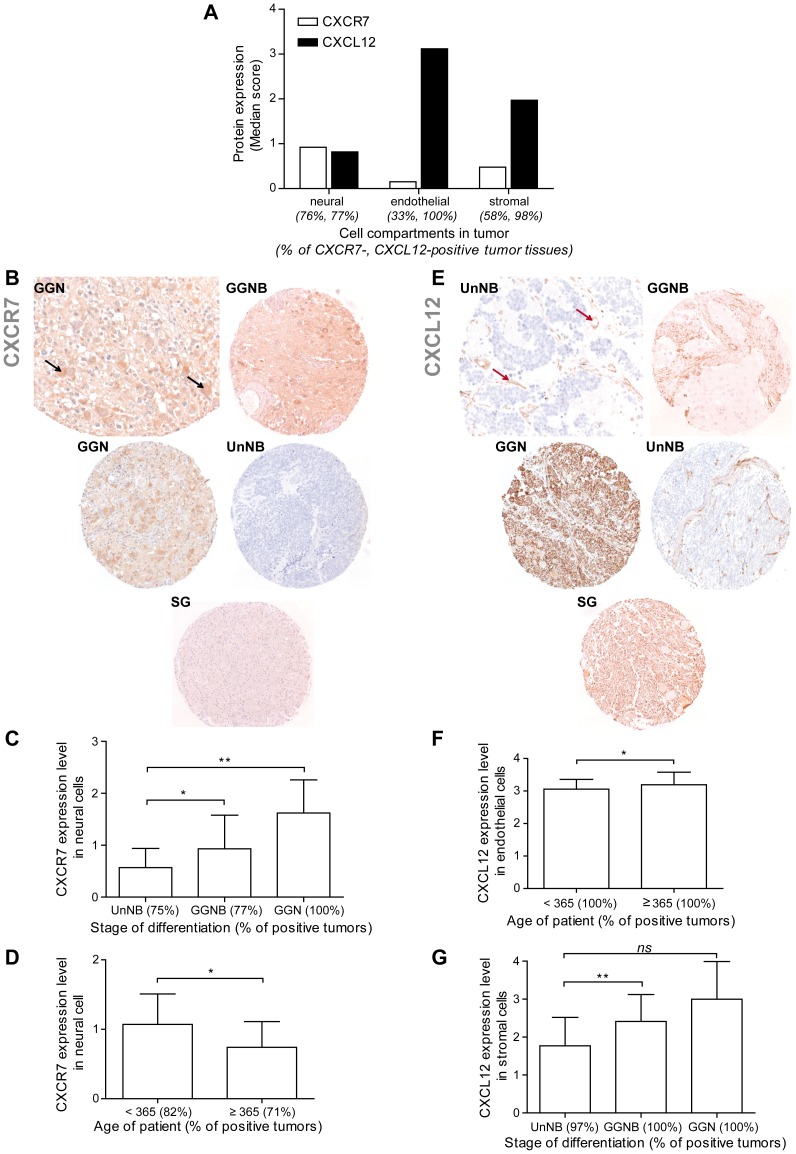
Expression of CXCR7, and its ligand CXCL12 in a NB TMA. (A) Semi-quantitative assessment of CXCR7 and CXCL12 expression levels in neural, endothelial and stromal cell compartments of NB primary tumors. Median score represents the average of the immunostaining score (0–4). Percentage (%) indicates percentage of CXCR7-or CXCL12-positive tumor tissues. (B) Immunohistochemical analysis of CXCR7 in undifferentiated tumor (UnNB), ganglioneuroblastoma (GGNB), ganglioneuroma (GGN) and control normal sympathetic ganglion (SG) tissues. Black arrow: ganglion cell. (C) CXCR7 expression levels (median score) in UnNBs, in differentiated tumor tissues, (D) and in tumors of patient according to the age of patient at diagnosis. (E) Immunohistochemical analysis of CXCL12 in UnNBs, GGNBs, GGNs and SG tissues. Red arrow: endothelial cell. (F) CXCL12 expression levels (median score) in the endothelial compartment of tumors according to the age of patient at diagnosis, (G) and in the stroma of UnNBs, GGNBs and GGNs. Student’s t-test: *p<0.05, **p<0.01.

A low CXCR7 expression (median score of 0.92) was observed in neural cell compartment in 76% of NB primary tumors (PTs), while no CXCR7 expression (all median scores <0.5) was detected in the vascular and stromal compartments of PTs, metastatic and control tissues (Figure 1A, Table 2). Thus, CXCR7 staining, albeit low, was generally localized in the neural compartment of NBs. Interestingly, CXCR7 expression did not correlate with NB grades (Table S1), but significantly enhanced with tumor differentiation stage (Figure 1B, 1C). Neural-associated CXCR7 staining score was significantly enhanced in differentiated tumors, such as ganglio-neuroblastomas (GGNBs) (median score of 0.93±0.65, p<0.05) and ganglioneuromas (GGNs) (median score of 1.62±0.64, p<0.01), when compared to undifferentiated NBs (UnNBs) (median score of 0.57±0.37). In particular, our data showed that CXCR7 staining was associated to tumor ganglion cells (black arrow) in GGNBs and GGNs, while no staining was observed in normal SG tissues. Moreover, CXCR7 expression increased in tumors from less than 1 year-old patients (Figure 1D), whom are known to present tumors with the potential to regress spontaneously, or to mature into benign matured tumors, such as GGNs [2]. Despite these observations, the TMA analyses did not allow to assign CXCR7 a statistically significant favorable prognosis value (data not shown).

**Table 2:** Expression of CXCR7 and CXCL12 in NB primary tumors, metastases and control tissues.

	Neural cells			Endothelial cells			Stromal cells		
	*Primary tumor*	*Metastasis*	*Control*	*Primary tumor*	*Metastasis*	*Control*	*Primary tumor*	*Metastasis*	*Control*
***CXCR7 expression***									
Positive tissues (%)	76	75	63	33	33	26	58	53	24
Number of cases	119	42	41	52	19	17	92	30	16
Median score	0.92	0.93	0.78	0.15	0.18	0.14	0.48	0.45	0.2
*p-value*		*ns*	*ns*		*ns*	*ns*		*ns*	*0.01*
***CXCL12 expression***									
Positive tissues (%)	77	92	70	100	100	100	98	98	93
Number of cases	121	52	46	156	56	65	153	55	61
Median score	0.82	1.04	0.59	3.12	3.13	3	1.97	1.93	1.98
*p-value*		*0.02*	*0.01*		*ns*	*0.01*		*ns*	*ns*

Control regroups normal adrenal gland and sympathetic ganglion tissues; Median score means average tumor score, as established by semi-quantitative analysis of the immunostaining; p-value (Student’s t-test) refers to primary tumor: ns means not significant, p<0.05 is considered significant, p≤0.01 is considered very significant.

#### CXCL12 is associated to the vascular and stromal structures of NBs

The CXCL12 ligand was strongly expressed in endothelial cells (red arrow) in all PTs (median score of 3.12), and highly associated to the stroma (median score of 1.97), while weakly expressed in the neural compartment (median score of 0.82) in NB PTs (Figure 1A, 1E, Table 2). In particular, tumor endothelial cells expressed higher levels of CXCL12 when compared to normal tissues (Table 2). Although, vascular CXCL12 expression was found independent of NB clinical stages (Table S1), it increased in tumors from patients over one year-old, whom are known to potentially present aggressive tumors [2] (Figure 1F). Even though high and similar CXCL12 levels were observed in the stromal compartment of tumors and controls (mean scores of 1.9, Table 2), ligand expression was further enhanced in the schwannian stroma of GGNBs, and, albeit not statistically significant, of GGNs (Figures 1E, 1G). In addition, chemokine expression, even low, was enhanced in the neuroblastic compartment of metastatic samples as compared to PTs, and in PTs as compared to controls (Table 2).

### Expression of the CXCR7 Receptor in NB Cell Lines

#### CXCR7 expression in N-, S-and I-type NB cell lines

**Figure 2:**
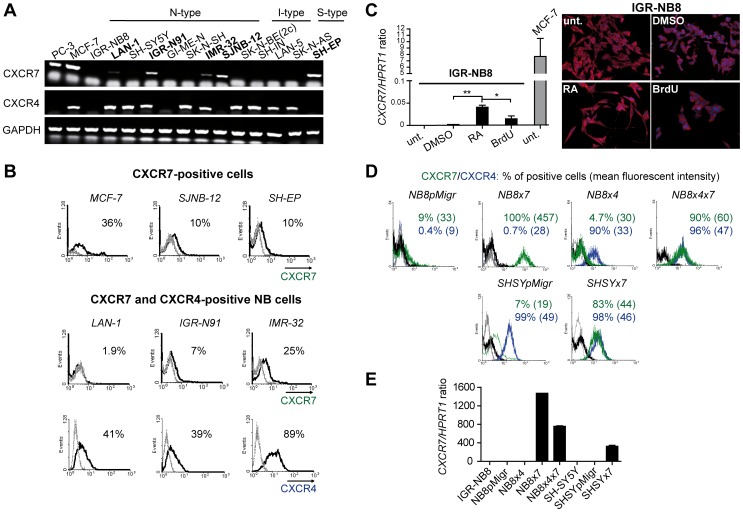
Expression of the two CXCL12 receptors in NB cell lines. (A) Qualitative RT-PCR analyses for *CXCR7* and *CXCR4* mRNA expression in a panel of N-, I-and S-type NB cell lines. *GAPDH* was used as gene of reference. The prostate cancer cell line PC-3 and the breast cancer cell line MCF-7 were used as positive controls for *CXCR7* expression. (B) Flow cytometry analyses of CXCR7 and CXCR4 cell surface expression in NB cell lines. Percent represents CXCR7-or CXCR4-positive cells. Grey line: cells stained without the primary Ab. Black line: cells stained with anti-CXCR7 or anti-CXCR4. (C) Semi-quantitative real-time PCR analyses of *CXCR7* mRNA expression level in the IGR-NB8 cell line after 3 days of treatment with either 10 µM RA or BrdU (left panel). Expression levels of *CXCR7* transcripts were calculated relatively to the level of the housekeeping gene *HPRTI*. Untreated (unt.) cells and DMSO-treated NB cells represented control conditions. Columns indicate results in triplicates, and were representative of two independent experiments. Error bars indicate S.D. Student’s t-test: *p<0.05, **p<0.01. Images (right panel) represent immunofluoresence staining of β_3_-tubulin (red) and DAPI (blue) in treated or untreated IGR-NB8 cells. (D) CXCR7, CXCR4, or a combination of the two CXCL12 receptors was overexpressed in the IGR-NB8 and the SH-SY5Y cell lines. As all vectors encoded for EGFP, transduced GFP-expressing NB cells were sorted by FACS to control transfection efficiency. Both NB8pMigr and SHSYpMigr cell lines represented control cells, transduced with the pMigr empty vector. Percent of CXCR7-and CXCR4-positive transduced cells are indicated as well as the mean fluorescent intensity (brackets) for CXCR7 and CXCR4 staining. Dark and grey lines: cells stained without anti-CXCR7 and anti-CXCR4, respectively; Green and blue lines: cells stained with anti-CXCR7 and anti-CXCR4, respectively. (E) Semi-quantitative real-time PCR analyses for *CXCR7* mRNA expression levels in NB transduced cell lines. Experiment was performed in triplicates. Error bars indicate S.D.

To corroborate our TMA analyses, we next assessed *CXCR7* expression by RT-PCR analyses in well-characterized NB cell lines harboring neuronal-like (N-type), glial/schwannian-like (S-type), or intermediate and undifferentiated (I-type) phenotype [1], [34]. In contrast to *CXCR4*, *CXCR7* expression was not detected in any analyzed I-type NB cell lines (SK-N-Be(2c), LAN-5, SH-IN), while 4/8 N-type cell lines (IGR-N91, LAN-1, IMR-32, SJN-B12) and 1/2 S-type cell line (SH-EP) expressed the receptor (Figure 2A). Moreover, poor CXCR7 surface expression was detected in RT-PCR-positive NB cells, while only IMR-32 cells harbored significant levels of both CXCL12 receptors (Figure 2B). Thus, our data suggested that, as observed in tumor tissues, CXCR7 expression may be linked to NB cell differentiated phenotype, as no CXCR7 expression was detected in the most undifferentiated NB cell lines.

#### CXCR7 expression may be associated with NB cell differentiation in vitro

As we observed a stronger CXCR7 expression in differentiated tumor tissues, we next investigated whether the receptor expression could be also associated with NB cell differentiation *in vitro*. Consequently, we induced CXCR7-negative NB cell lines to differentiate *in vitro*, by using all-trans retinoic acid (RA) and bromodeoxyuridine (BrdU) [38], [42], [43]. As N-and I-type NB cell subtypes have been shown to progress towards neuronal or glial/schwannian fate upon RA or BrdU treatment, respectively [44], N-type IGR-NB8 and SH-SY-5Y cells, as well as I-type SK-N-Be(2c) cells were exposed to 10 µM of either differentiation agent for 30 days. NB cell morphology changes appeared as early as 3 days after either RA or BrdU exposure (right panel, Figure 2C), and persisted during all the differentiation induction experiment (Figure S1A), as previously observed [44]. Moreover, treated NB cells elicited global reduced proliferation, without enhanced apoptosis, further confirming an induced differentiation of treated NB cells *in vitro* (Figure S1B and S1C). As shown in Figure 2C (left panel), *CXCR7* expression was induced in IGR-NB8 cells after 3 days of RA treatment (p<0.01), whereas its expression was weakly detectable upon BrdU exposure. Similar RA-induced CXCR7 expression pattern was also detected in the SH-SY5Y and the SK-N-Be(2c) cell lines (Figure S2A, S2B). These data thus suggested that induction of *CXCR7* expression, albeit weak, preferentially occurred when NB cells underwent neuronal rather than glial/schwannian differentiation. Addition of CXCL12 together with RA or BrdU treatment did not further increase receptor expression in treated NB cells (Figure S2B). However, induced CXCR7 protein levels might be too low, or post-translationally modified, as they could be neither detected at the cell surface (FACS analyses), nor in the intracellular space of treated NB cells (Immunofluorescence assays), by both anti-CXCR7 antibodies used in this study (data not shown).

#### Ectopic expression of CXCR7 in NB cell lines

Although CXCR7 was found in a minority of NB cell lines as compared to CXCR4, its expression was however detected in some CXCR4-expressing NB cells. Consequently, we next focused on the individual roles and functional interactions between CXCR7 and CXCR4 in NB. To that extent, individual CXCR7, CXCR4 or combined receptors were overexpressed in the CXCR4/CXCR7-negative IGR-NB8 cell line (respectively NB8×7, NB8x4 and NB8×4×7 cell lines). CXCR7 was also ectopically overexpressed in the SH-SY5Y cell line (SHSY×7 cells), which expresses high endogenous CXCR4 levels (Figure 2D). Of note, a decrease of CXCR7 surface expression was observed in SHSY×7 cells (mean fluorescent intensity of 44) as compared to NB8×4×7 cells (mean fluorescent intensity of 60), while similar CXCR4 surface expression was detected in these two cell lines. Different *CXCR7* expression levels in these two double receptor-expressing cell lines were confirmed by semi-quantitative RT-PCR analyses (Figure 2E). Thus, the two CXCR7/CXCR4-expressing transduced cell lines harbored variable relative expression levels of the two CXCL12 receptors.

### CXCL12 and CXCL11 Induce ERK1/2, but not Akt Pathway Activation in CXCR7-expressing NB Cells

**Figure 3:**
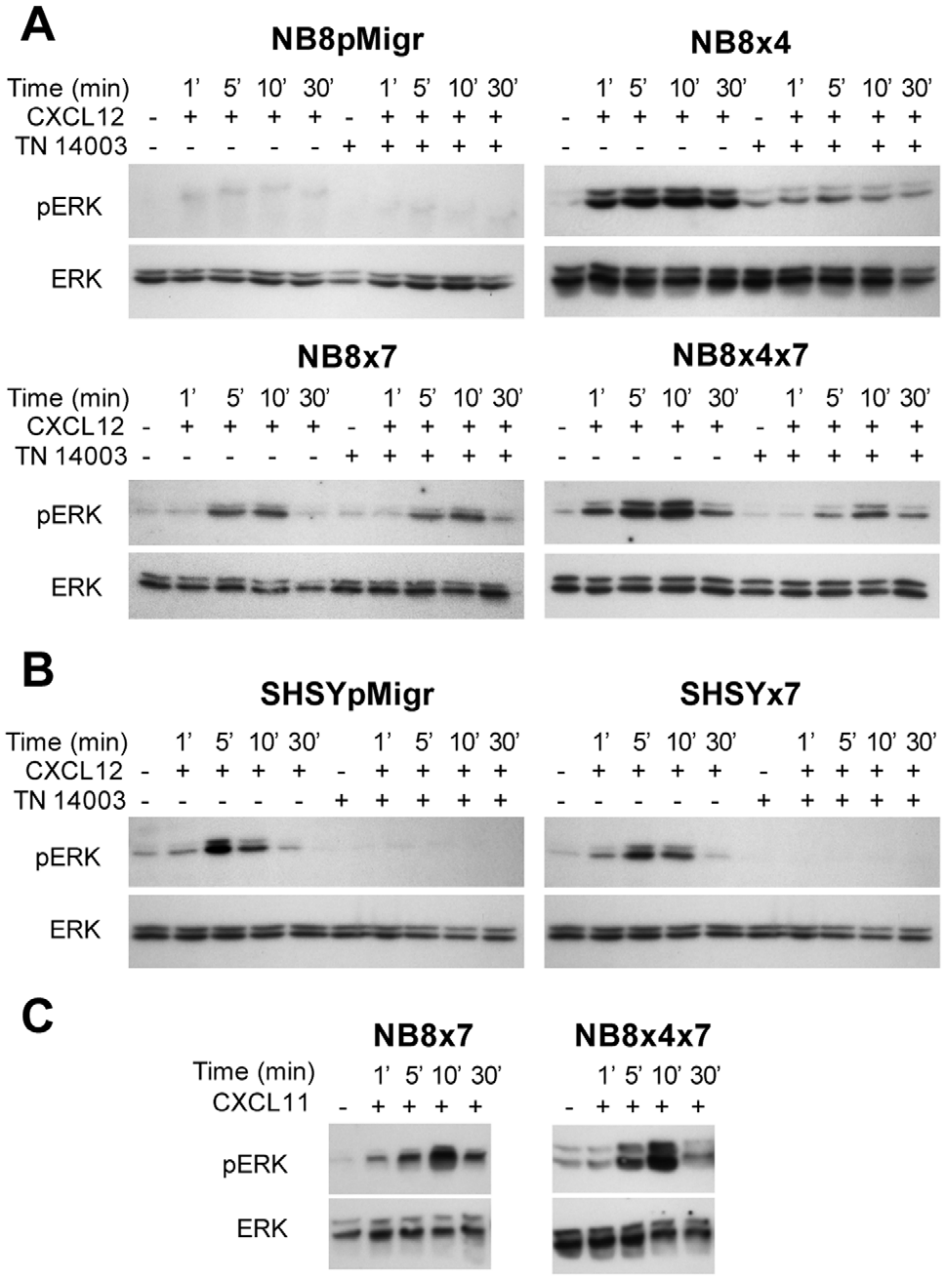
ERK1/2 activation in NB cell lines. Immunobloting of phospho-ERK (pERK) and total ERK (ERK) in transduced cells, treated with (A, B) 100 ng/ml human recombinant CXCL12, in presence or in absence of 1 µM of the CXCR4 blocker TN14003, (C) 100 ng/ml human recombinant CXCL11.

As CXCR7/CXCL12-mediated ERK1/2 activation was detected in various models [22], [23], [45], [46], we next assessed ERK1/2 cascade activation in NB transduced cells, in response to either CXCL12 or CXCL11 ligand. We showed herein that ERK1/2 was activated in CXCR4-and CXCR7-transduced NB cells after CXCL12 stimulation, indicating that CXCR4 and CXCR7 were both able to activate downstream pathways in response to their common ligand (Figure 3A). Interestingly, constant ERK1/2 activation was maintained until 30 min after CXCL12 stimulation in NB8x4 cells, whereas enhanced intensity after 5 and 10 min, followed by a signal decrease was observed in the NB8×7 and NB8x4×7 cell lines. These data indicated that CXCR7 might interact with CXCR4/CXCL12-mediated signaling. Moreover, ERK1/2 activation was lost in CXCR4-expressing NB8×4 cells upon addition of the specific CXCR4 inhibitor (TN14003), further confirming that this activation was specific and restricted to the CXCR4/CXCL12 axis in those cells. As ERK1/2 activation was not completely inhibited by TN14003 treatment in NB8×4×7 cells, it further suggested that ERK1/2 activation was partially mediated by the CXCR7/CXCL12 axis in CXCR7/CXCR4-expressing NB8×4×7 cells. In parallel, CXCR7 only slightly weakened CXCR4/CXCL12-induced ERK1/2 activation in the other double receptor-positive SHSY×7 cell line, and was not able to signal via ERK1/2 upon addition of both CXCL12 and CXCR4 inhibitor (Figure 3B). In addition, CXCR7 was also able to activate ERK1/2 cascade in NB8×7 and NB8×4×7 cells upon CXCL11 engagement (Figure 3C), but not in SHSY×7 cells (data not shown). These data thus suggested that the two CXCR7/CXCR4-expressing SHSY×7 and NB8x4×7 cell lines differently responded to CXCL12 and CXCL11.

Looking for additional downstream effectors of CXCR7 and CXCR4 receptors, we also evaluated Akt activation upon stimulation of NB cells with either CXCL12 or CXCL11 chemokine ligand [10], [47]. However, neither the CXCR4/CXCL12 nor the CXCR7/CXCL12/CXCL11 axes were able to activate Akt in transduced NB cells (Figure S3).

### In Contrast to CXCR4, CXCR7 Alters NB Growth *in vitro*

**Figure 4:**
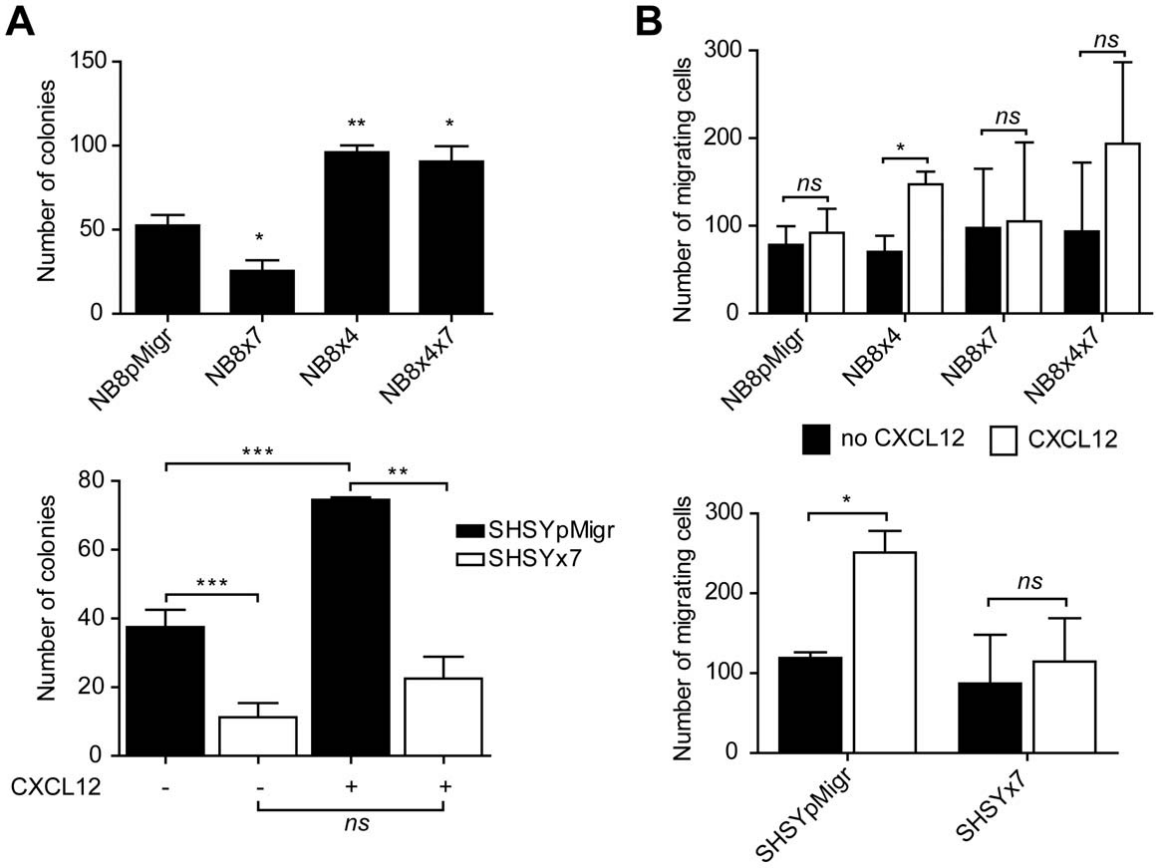
Impact of CXCR7 on NB growth and migration in vitro. (A) Clonogenic growth of NB transduced cell lines was evaluated in a soft agar assay, after at least two weeks of incubation at 37°C. Columns: mean values ± SEM of two independent experiments. When stipulated, 100 ng/mL CXCL12 were added to the culture medium. (B) Chemotaxis of transduced NB cells toward 100 ng/ml CXCL12. Columns: mean values ± SEM of at least two independent experiments. Student’s t-test was used for all experiments: *p<0.05, **p<0.01, ***p<0.001.

As the CXCR7 receptor was shown here to activate growth-regulating pathway such as ERK1/2 cascade, we next explored the role of CXCR7, and analyzed the relative contribution of the two CXCL12 receptors in mediating NB growth *in vitro*. Our data showed that the presence of CXCR4 significantly enhanced NB8×4 and NB8×4×7 cell clonogenic abilities, while CXCR7 expression in NB8×7 cells resulted in significantly decreased colony number, when compared to the NB8pMigr cell line, and in absence of the ligand (Figure 4A, upper panel). Interestingly, the presence of CXCR7 also significantly decreased the number of colonies derived from the SHSY×7 cell line, in absence and in presence of CXCL12 (Figure 4A, lower panel). Addition of the ligand markedly increased the clonogenic capacity of the CXCR4-positive SHSYpMigr cell line, without affecting that of SHSY×7 cells. These data thus suggested that CXCR7 affected the CXCR4-mediated SHSY×7 growth, but not that of NB8×4×7 cells *in vitro*.

### CXCR7 Impairs CXCR4/CXCL12-mediated NB Chemotaxis

The CXCR7/CXCL12 axis has been proposed to induce tumor cell migration in various cancer models [10], [46], [48]. Therefore, we next evaluated the impact of CXCL12 binding to CXCR7 on NB chemotaxis *in vitro*. No migration toward the ligand CXCL12 was observed using CXCR7-expressing NB8×7 cells and CXCR7/CXCR4-expressing NB8×4×7 and SHSY×7 cells (Figure 4B). In contrast, the presence of CXCL12 significantly enhanced motility of CXCR4-expressing cells (NB8×4 and SHSYpMigr cells), as previously described [16]. Thus, these data showed that the CXCR7/CXCL12 pair could not stimulate NB chemotaxis, and further suggested CXCR7 as a negative regulator of CXCR4 signaling, as it altered CXCR4/CXCL12-mediated chemotaxis of NB cells *in vit*ro.

### CXCR7 Abrogates Subcutaneous NB Growth

**Figure 5:**
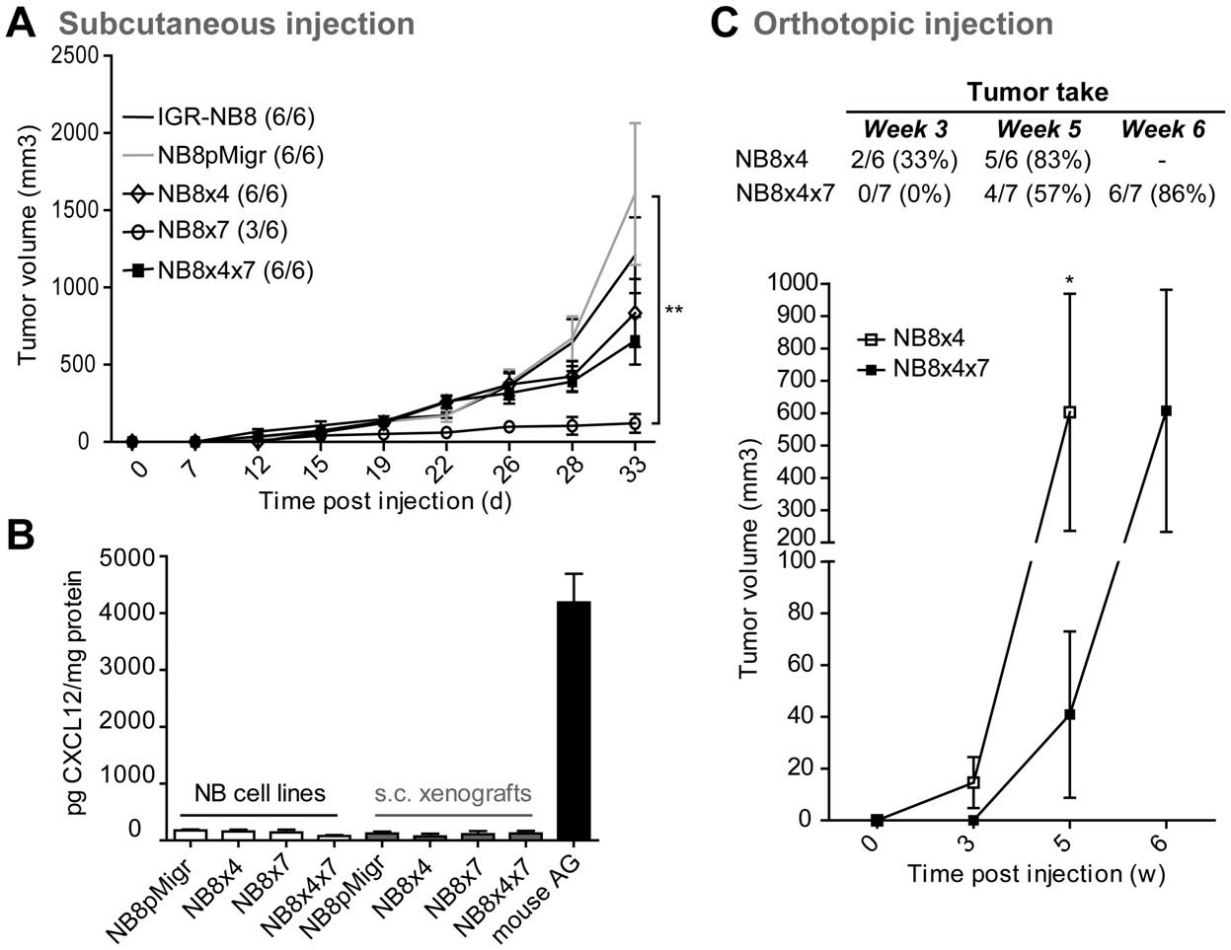
Impact of CXCR7 on NB growth in vivo. (A) *In vivo* tumor take (number of sites with tumor/total sites) and growth (mean tumor volume ± SEM) after *s.c* implantation of transduced NB cells in nude mice. Two-way ANOVA: **p<0.01. (B) Production of CXCL12 was measured by ELISA in transduced NB cell lines, and derived *s.c* tumors. Normal nude mouse adrenal gland (AG) tissue was used as positive control. Results are expressed in triplicates as pg of CXCL12 per mg of extracted protein. Error bars indicate S.D. of triplicates. (C) *In vivo* orthotopic implantation of NB8×4 and NB8×4×7 cell lines in nude mice. Upper panel: tumor take represented as fraction and percentage of tumor-bearing mice from week 3 to week 6 after NB cell implantations. Lower panel: kinetics of tumor volume. Mann-Whitney test: *p<0.05.

We next addressed the impact of CXCR7 on NB growth *in vivo*, and particularly its ability to regulate and/or impair the CXCR4-mediated effects. In subcutaneous conditions, overall tumor take was reduced in the group of mice engrafted with NB8×7, as only 3/6 sites presented a tumor versus 6/6 sites for the other groups (Figure 5A). The volume of NB8×7 cell-derived tumors was also significantly reduced, as compared to that of derived from the control NB8pMigr cell line. Supporting our *in vitro* observations, these data further suggested CXCR7 as a critical player in NB growth regulation. However, H&E staining analyses did not reveal particular differentiation area on paraffin-embedded sections of NB8×7 cell-derived xenografts (data not shown). In addition, growth of NB8 cell-derived tumors was not significantly affected by the presence of CXCR4 alone, nor in association with CXCR7 in such *in vivo* conditions.

To evaluate a putative functionality of the CXCL12 ligand in CXCR7-mediated effect in our heterotypic mouse model, we measured the concentration of CXCL12 in *s.c* xenografts, and associated NB transduced cell lines (Figure 5B). As CXCL12 was highly produced in mouse adrenal gland tissues [16], production levels of the chemokine in such tissues were used as positive control. CXCL12 production in NB cell lines and xenografts was low (mean concentration of CXCL12<180 pg/mg of protein) and did not significantly vary between either cell lines or derived tumors, suggesting that the CXCR7-mediated anti-proliferative effect is unlikely due to the presence of its ligand CXCL12 in such *in vivo* conditions.

### CXCR7 Delays CXCR4-mediated Proliferative Effect in Orthotopic Conditions

As we showed herein that CXCR7 enabled NB growth reduction in a heterotypic mouse model (Figure 5A), and altered CXCR4/CXCL12-mediated NB migration (Figure 4B), we further evaluated the extent to which CXCR7 would affect both the *in vivo* CXCR4-mediated growth promoting effect and NB dissemination in a orthotopic environment. To that purpose, NB8×4 and NB8×4×7 cells were orthotopically implanted in mouse adrenal gland, as previously described [16], and tumor growth was evaluated by echography for 6 weeks (Figure 5C). At week 3, 33% of animals engrafted with NB8×4 cells developed a tumor, whereas no tumors were detected in the NB8×4×7 group. Interestingly, the volume of NB8×4×7 cell-derived tumors was significantly reduced as compared to that of the NB8×4 group at week 5 (p<0.05), suggesting that CXCR7 significantly affected tumor take of tumors derived from CXCR4-positive NB8×4×7 cells. Due to excessive tumor volume in the NB8×4 group, mice had to be sacrificed at earlier time point (week 5) than those injected with the NB8×4×7 cell line (week 6). At week 6, 6/7 mice in the NB8×4×7 group developed tumors, with volume similar to those observed at week 5 with the NB8×4 group. No macroscopic metastases were observed in either group.

## Discussion

The CXCR4/CXCL12 axis has been largely shown to participate in tumor development and progression [9], [16]. Although several hypotheses on the role of CXCR7 and its possible interaction with CXCR4 have been proposed in different tumor systems, the functional implication of the global CXCL12/CXCR7/CXCR4 axis in NB remains unknown. Our TMA analyses revealed that CXCR7 expression was generally weak in primary NB of all stages and in metastatic tissues. Moreover, in contrast to breast, lung and hepatocellular carcinomas, CXCR7 was not expressed in NB vasculature but rather preferentially associated to its neural compartment [30], [49]. Interestingly, CXCR7 expression was associated to mature neural cells, such as ganglion cells, in stroma-rich GGNB and GGN tumors, which are associated to a favorable prognosis [2]. However, no statistically significant favorable prognosis value could be assigned to CXCR7. The absence of significance may be explained by the low and variable levels of CXCR7 expression in heterogeneous NB tissues. Nonetheless, the particular CXCR7 expression pattern on mature cells suggested an implication/association of the CXCR7 receptor with NB differentiation.

Interestingly, TMA analyses also revealed a strong CXCL12 expression in endothelial and stromal cells in tumors, suggesting a paracrine role of the chemokine in NB. In particular, a putative implication of the ligand in NB angiogenesis is likely, as already reported in the context of ovarian and colon cancers [50], [51].

The pattern of CXCR4 expression in NB has been already shown to be related to high stage disease, including non-metastatic stage 3 and metastatic stage 4 NBs [52]. As the two receptors elicited specific expression patterns in NB tissues, our TMA analyses suggest a complex contribution of the CXCR7 and CXCR4 receptors in NB pathogenesis, which may be tightly modulated by a permanent cross-talk with their common ligand CXCL12, highly produced by tumor microenvironment.

Screening of NB cell lines by RT-PCR analyses revealed specific *CXCR7* expression in N-type and S-type NB cell lines, rather than in the most undifferentiated I-type NB cell lines [53], suggesting an association of CXCR7 expression with neuronal-and/or glial/schwannian NB cell phenotype. A link between CXCR7 expression and cell differentiation phenotype has already been reported in immune cells. CXCR7 expression was indeed proposed to correlate with dendritic cell maturation, and described as a potential maker of differentiating memory B cells [54]. Moreover, CXCR7 expression has been also shown to drastically increase in FCS-induced differentiation of glioma cells *in vitro* [45]. A weak induced *CXCR7* expression was observed in NB cells exposed to RA, but not to BrdU, suggesting that CXCR7 may be associated with neuronal rather than glial differentiation. These *in vitro* analyses correlated our TMA data showing CXCR7 staining in tumor ganglion cells, rather than in schwannian stroma. However, CXCR7 could be neither detected at the surface, nor in the intra-cellular space of NB cells during all the differentiation induction experiment. These observations suggest that receptor expression may be modulated by potential post-translational modifications, or that putative induced-protein expression is too low to be detected by antibodies used in this study. In addition, exogenous CXCR7 did not induce, by its own, phenotypic changes in the slow proliferating-tumors in our heterotypic mouse model. Indeed, no ganglion-like cells and no differentiating neuroblasts were detected in NB8x7-derived xenografts. Therefore, further investigation will be necessary to determine the intimate link between CXCR7 expression and NB differentiation process.

Although specifically expressed in differentiated and matured tumors, CXCR7 was also detected in a weak percentage of tumor cells in tissues, independently of NB clinical stages. As CXCR4 is largely expressed in high grade NBs, co-expression of the two CXCL12 receptors in tumor tissues is then likely. Screening of NB cell lines confirmed such hypothesis by showing co-expression of CXCR7 and CXCR4 in some NB cells, as described elsewhere [55]. Consequently, we next examined the role of CXCR7 in NB, and particularly its relation with CXCR4. CXCR7, like CXCR4, was able to induce downstream signaling pathway on its own. However, co-expression of the two receptors in NB cells led to a modulation of ERK1/2 activation in presence of CXCL12, demonstrating a functional interaction between CXCR7 and CXCR4 in NB, as described in other models [56]. However, induction of ERK activation by either CXCL12 or CXCL11 appeared to be cell line-dependent, as NB8x4x7 and SHSYx7 cells responded differently towards these chemokines. Such discrepancy may result from CXCR4 endogenous expression levels, from variable CXCR7 exogenous levels detected in these NB cell lines, or suggests that CXCR7 may signal through pathways other than ERK1/2 cascade in NB cells.

In contrast to CXCR4, CXCR7 alone significantly decreased *in vitro* NB cell clonogenic potential, in absence of CXCL12. Furthermore, CXCR7, by its own, also significantly reduced subcutaneous growth of NB cell-derived tumors, independently of its ligand. Such data are further supported by a recent study showing that proliferation of CXCR7-positive glioma cells may not be affected by CXCL12 [45]. In addition, a ligand-independent role for CXCR7 has been also demonstrated in a prostate cancer model [57]. Therefore, CXCR7 and CXCR4 might individually display opposite ligand-independent-mediated functions in NB.

Significant *in vitro* alterations of NB clonogenicity were noted in the SHSY×7 cell line expressing both CXCR7 and CXCR4 receptors, in presence or in absence of CXCL12. In an orthotopic and CXCL12-producing environment, CXCR7 was not able to suppress the growth of large, established tumors, as growth curve slopes of both NB8×4 and NB8×4×7 cell-derived tumors were similar in the exponentially tumor growth phase. However, in such environment and in contrast to *s.c.* conditions, presence of CXCR7 clearly resulted in a delayed tumor take of CXCR4/CXCR7-positive cell-derived tumors, as compared to NB8×4 cell-derived tumors. Consequently, our observations suggest a critical role of CXCR7 in regulating CXCR4-mediated effects in NB, and underline the essential impact of a particular microenvironment on NB cell behavior, as mentioned in our previous study [16].

CXCR7 alone did not favor migration of NB cells toward CXCL12, in contrast to CXCR4. Conversely, a very recent study reported that CXCR7 enhanced chemotaxis of CXCR7-expressing NB cells in presence of CXCL12-producing mesenchymal stromal cells [55]. However, it was not clear whether additional factors released by stromal cells were required for truly activating NB chemotaxis. More interestingly, CXCR7 significantly altered the CXCR4-mediated chemotaxis of NB8×4×7 and SHSY×7 cells toward CXCL12, further supporting a negative regulation of CXCR4 by CXCR7.

It has been recently proposed that CXCR7 may control CXCL12 distribution by sequestrating the ligand present in the extracellular space. In such model, CXCR7 enables establishment of effective CXCL12 gradients, thus resulting in increased responsiveness of CXCR4 signaling and chemotaxis in response to these gradients [58], [59]. In that context, CXCR7 has been clearly demonstrated to control cell migration in a zebrafish model, and to regulate migratory advantage provided by CXCR4 in CXCR4/CXCR7-expressing primary T cells and tumor cells [32], [59], [60], [61]. However in the present study, co-expression of CXCR7 with CXCR4 apparently did not favor metastatic dissemination of a non-aggressive IGR-NB8 cell line *in vivo*, as no metastases were detected after orthotopic implantation of NB8×4×7 cells. In the other hand, ligand-scavenging effect attributed to CXCR7 has been also proposed to limit acute CXCR4/CXCL12-mediated signaling. Indeed, Hernandez *et al* suggested that CXCR7 scavenging function might impair CXCR4-induced breast tumor cell invasion, by down-regulating CXCR4/CXCL12-mediated metalloproteinase-12 production [33]. As altered CXCR4/CXCL12-mediated chemotaxis and growth were observed upon co-expression of the two CXCL12 receptors in NB cells, such ligand scavenging role for CXCR7 appears likely, and may enable a negative regulation of CXCR4/CXCL12-mediated fonctions in NB.

CXCR7 was also reported to act as a co-receptor for CXCR4. Combined CXCR4/CXCR7 expression has been detected in primary human tumors and tumor cell lines [45], [62], [63]. Structural association of the two receptors has been shown to affect CXCR4/CXCL12-mediated G-protein signaling [64]. As ERK cascade activation was modulated in CXCR4/CXCR7-expressing NB cells as compared to CXCR4-expressing NB cells, putative heterodimerization of the two receptors in NB cells is thus possible. Moreover, it has been hypothesized that CXCR7, once engaged in heterodimers with CXCR4, may also negatively regulate CXCR4 functions through an allosteric mechanism (independently of CXCL12) [64]. These observations further support our data showing an alteration of the CXCR4-mediated growth promoting effect *in vitro* in SHSYx7 cells, particularly in absence of CXCL12. Several lines of evidence have suggested that chemokine receptor homo-or heterodimerization activates distinct signaling pathways, and thus distinct biological responses [65]. As the two CXCL12 receptors may form heterodimers as efficiently as homodimers [64], tight regulation of CXCR4 and CXCR7 expression may enable variable conformations of the two receptors at cell membrane, and may thus lead to activation of distinct signaling [66], [67]. Such hypothesis may explain the heterogeneity of responses observed *in vitro*, using the two transduced NB cell lines expressing variable CXCR7/CXCR4 receptor expression levels.

To conclude, our data reveal distinct functional roles for the two CXCL12 receptors in NB. While CXCR4 favors NB growth and chemotaxis, CXCR7 reduces tumor growth and may be associated to less aggressive stages of the disease. Our data clearly show anti-tumorigenic properties for the CXCR7 receptor in NB, as CXCR7 was able to affect *in vitro* migration-promoting effect mediated by the CXCR4/CXCL12 axis, and to delay orthotopic tumor take of CXCR4-positive NB cells. Taken together our observations suggest that CXCL12-induced responses may result from a direct or indirect cross-talk between CXCR7 and CXCR4, which may be tightly modulated by receptor expression and by a particular ligand-producing microenvironment. However, whether CXCR7 may modulate CXCR4 signaling as a result of their heterodimerization or/and by scavenging the ligand will need further investigation. Nonetheless, a putative cross-talk between the two CXCL12 receptors may give clues to elucidate the original and complex CXCR7/CXCR4/CXCL12 distribution and functions in NB pathogenesis.

## Supporting Information

Figure S1 **Characteristics of NB cells after RA or BrdU treatment in vitro.** (A) SK-N-Be(2c) cells were exposed to either 10 µM all-trans Retinoic Acid (RA) or 5-bromo-2-deoxyuridine (BrdU) for 3 and 30 days. Images illustrate immunofluoresence staining of β_3_-tubulin (red) and DAPI (blue). RA-treated NB cells elaborated enhanced neuritic processes and proliferated by forming interconnected cell clumps, while BrdU-treated cells presented glial-like morphology, with large flat cytoplasm and enhanced surface adherence. Morphological changes were not detected in untreated (unt.), nor in DMSO-treated control cells. (B) Growth of SK-N-Be(2c) (left panel) and IGR-NB8 (right panel) cells was followed upon treatment with either differentiation agent for 96 h. Columns represent OD mean ± SEM of two independent experiments. As previously described [44], RA enhanced SK-N-Be(2c) cell growth for 72 h, as compared to cells exposed to DMSO, before inducing a growth arrest in those cells at 96 h. Proliferation of IGR-NB8 cells already slowed down after 72 h of RA treatment, as compared to DMSO-treated control cells. BrdU-treatment induced a reduction of both SK-N-Be(2c) and IGR-NB8 cell growth, as compared to untreated cells. (C) Apoptosis was measured by detection of the sub-G_1_ apoptotic cell using the PI staining method [68]. Such assay was performed after 7 day-treatment with either 10 µM RA or BrdU. Treatment of NB cells with 1 µg/ml doxorubycin (Dox) for 48 h was used as positive control. A slight induction of mortality was noted for the SK-N-Be(2c) cell line when treated with RA, which was also previously reported [44], while no effect was observed upon treatment with BrdU, as compared to control cells. None of the treatments induced apoptosis of IGR-NB8 cells.
(TIF)

Figure S2 ***CXCR7*** **mRNA expression levels upon differentiation of NB cells in vitro.** (A) Semi-quantitative real-time PCR analyses of *CXCR7* mRNA expression levels upon treatment of SH-SY5Y cells with 10 µM RA or BrdU for 3 days. (B) The SK-N-Be(2c) and the IGR-NB8 cell lines were treated with 10 µM RA. Untreated cells (unt.) or cells exposed to DMSO were used as controls. When stipulated, 100 ng/mL CXCL12 were added to the culture medium. Expression levels of *CXCR7* transcripts were calculated relatively to the level of the housekeeping gene *HPRTI*. The breast cancer cell line MCF-7 and the prostate cancer cell line PC-3 were used as positive controls for *CXCR7* expression. Columns indicate results in triplicates and were representative of two independent experiments. Error bars indicate S.D. Student’s t-test: *p<0.05, **p<0.01.
(TIF)

Figure S3 **Akt pathway activation in NB cell lines.** Immunobloting of phospho-Akt (pAkt) and total Akt (Akt) in NB transduced cells, stimulated with (A) 100 ng/ml CXCL12, or (B) 100 ng/ml CXCL11 at indicated time points. NB transduced cells were also treated with 10 ng/ml IGF-1 for 1 h, as positive control [69].
(TIF)

Table S1 **Expression of CXCR7 and CXCL12 in NB clinical groups.** CXCR7 and CXCL12 expression, as associated to neural, endothelial and stromal cell compartments, were measured in INSS neuroblastoma clinical groups.
(DOC)
