# Supplementary figures and images for: Involvement of the CXCR7/CXCR4/CXCL12 Axis in the Malignant Progression of Human Neuroblastoma
Source: PLoS One. 2012 Aug 20;7(8):e43665. doi: 10.1371/journal.pone.0043665 (PMC3423387; doi:10.1371/journal.pone.0043665)

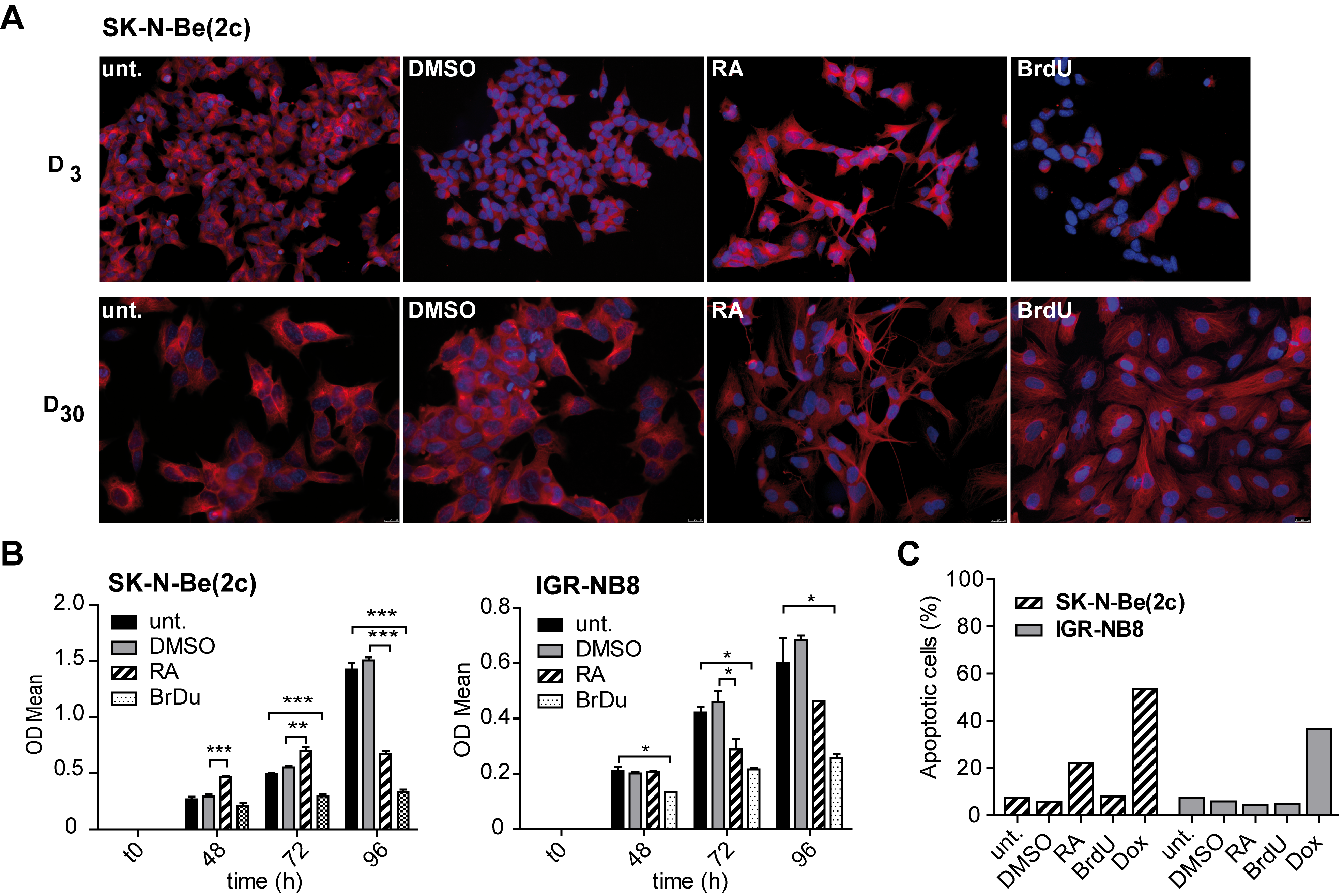

Supplement: Figure S1 — Characteristics of NB cells after RA or BrdU treatment in vitro. (A) SK-N-Be(2c) cells were exposed to either 10 µM all-trans Retinoic Acid (RA) or 5-bromo-2-deoxyuridine (BrdU) for 3 and 30 days. Images illustrate immunofluoresence staining of β3-tubulin (red) and DAPI (blue). RA-treated NB cells elaborated enhanced neuritic processes and proliferated by forming interconnected cell clumps, while BrdU-treated cells presented glial-like morphology, with large flat cytoplasm and enhanced surface adherence. Morphological changes were not detected in untreated (unt.), nor in DMSO-treated control cells. (B) Growth of SK-N-Be(2c) (left panel) and IGR-NB8 (right panel) cells was followed upon treatment with either differentiation agent for 96 h. Columns represent OD mean ± SEM of two independent experiments. As previously described [44], RA enhanced SK-N-Be(2c) cell growth for 72 h, as compared to cells exposed to DMSO, before inducing a growth arrest in those cells at 96 h. Proliferation of IGR-NB8 cells already slowed down after 72 h of RA treatment, as compared to DMSO-treated control cells. BrdU-treatment induced a reduction of both SK-N-Be(2c) and IGR-NB8 cell growth, as compared to untreated cells. (C) Apoptosis was measured by detection of the sub-G1 apoptotic cell using the PI staining method [68]. Such assay was performed after 7 day-treatment with either 10 µM RA or BrdU. Treatment of NB cells with 1 µg/ml doxorubycin (Dox) for 48 h was used as positive control. A slight induction of mortality was noted for the SK-N-Be(2c) cell line when treated with RA, which was also previously reported [44], while no effect was observed upon treatment with BrdU, as compared to control cells. None of the treatments induced apoptosis of IGR-NB8 cells. (TIF) [file pone.0043665.s001.tif]

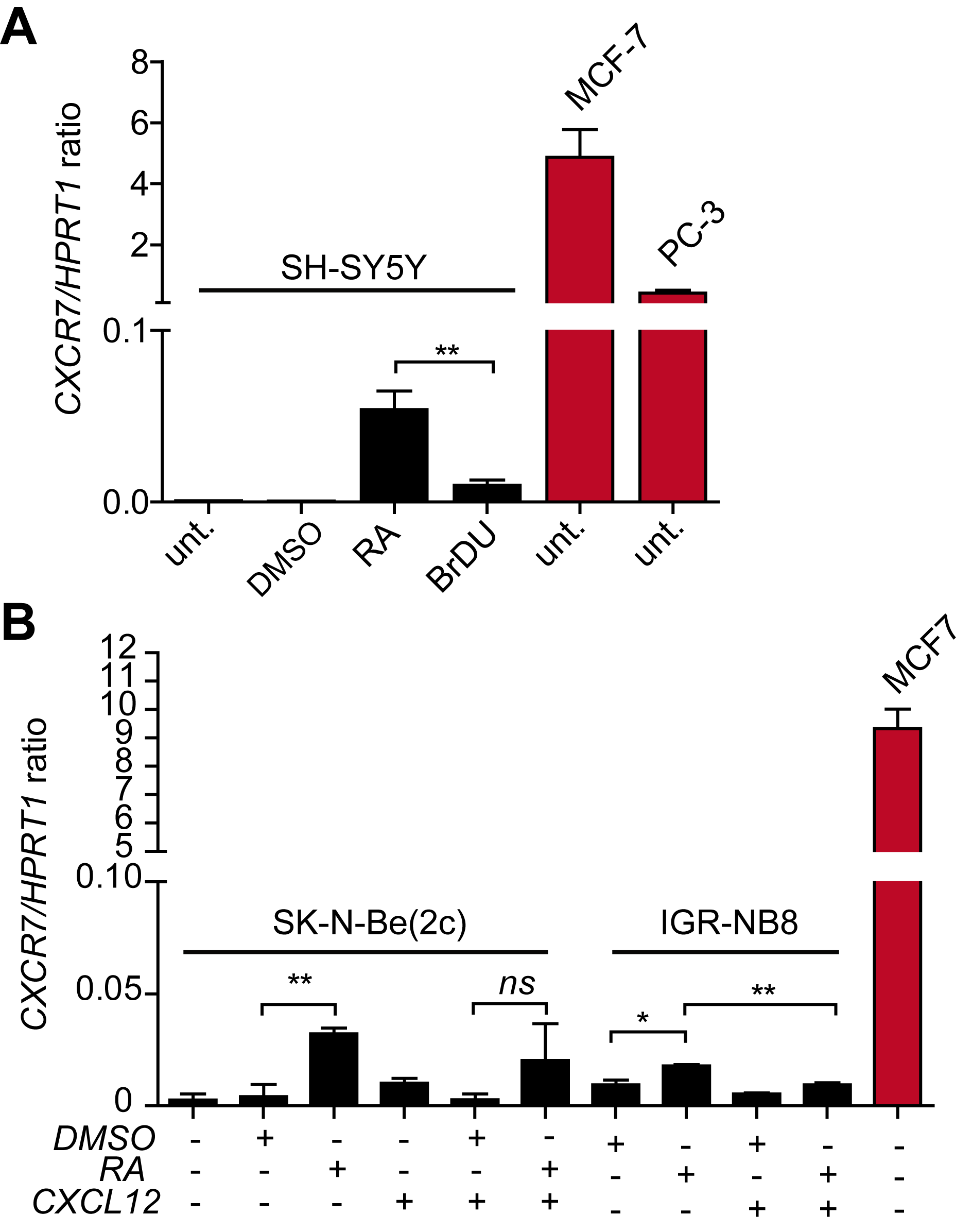

Supplement: Figure S2 — CXCR7 mRNA expression levels upon differentiation of NB cells in vitro. (A) Semi-quantitative real-time PCR analyses of CXCR7 mRNA expression levels upon treatment of SH-SY5Y cells with 10 µM RA or BrdU for 3 days. (B) The SK-N-Be(2c) and the IGR-NB8 cell lines were treated with 10 µM RA. Untreated cells (unt.) or cells exposed to DMSO were used as controls. When stipulated, 100 ng/mL CXCL12 were added to the culture medium. Expression levels of CXCR7 transcripts were calculated relatively to the level of the housekeeping gene HPRTI. The breast cancer cell line MCF-7 and the prostate cancer cell line PC-3 were used as positive controls for CXCR7 expression. Columns indicate results in triplicates and were representative of two independent experiments. Error bars indicate S.D. Student’s t-test: *p<0.05, **p<0.01. (TIF) [file pone.0043665.s002.tif]

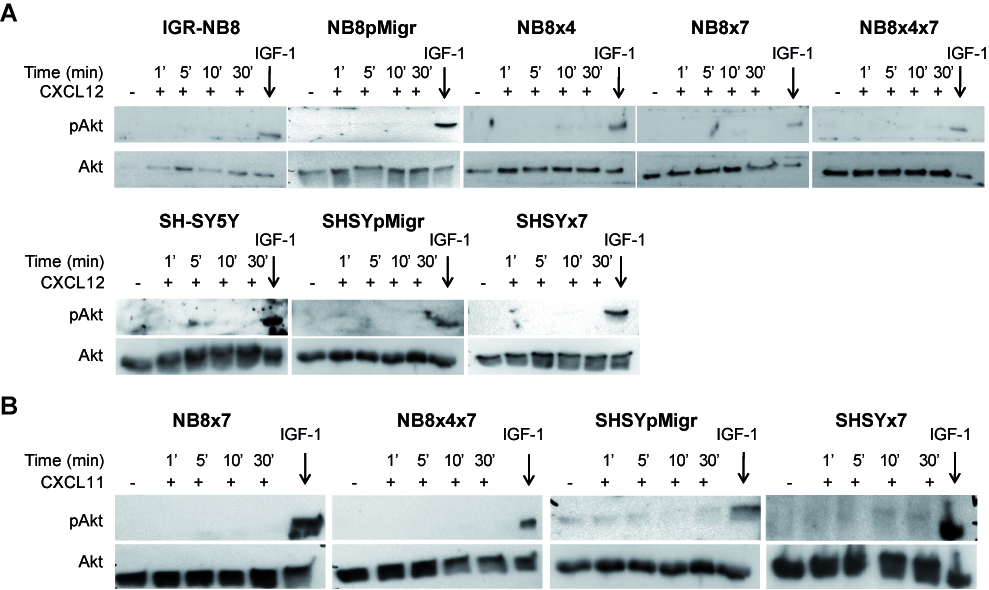

Supplement: Figure S3 — Akt pathway activation in NB cell lines. Immunobloting of phospho-Akt (pAkt) and total Akt (Akt) in NB transduced cells, stimulated with (A) 100 ng/ml CXCL12, or (B) 100 ng/ml CXCL11 at indicated time points. NB transduced cells were also treated with 10 ng/ml IGF-1 for 1 h, as positive control [69]. (TIF) [file pone.0043665.s003.tif]
