# Supplementary material for: Involvement of the CXCR7/CXCR4/CXCL12 Axis in the Malignant Progression of Human Neuroblastoma
Source: PLoS One. 2012 Aug 20;7(8):e43665. doi: 10.1371/journal.pone.0043665 (PMC3423387; doi:10.1371/journal.pone.0043665)
Supplement: Table S1 — Expression of CXCR7 and CXCL12 in NB clinical groups. CXCR7 and CXCL12 expression, as associated to neural, endothelial and stromal cell compartments, were measured in INSS neuroblastoma clinical groups. (DOC) [file pone.0043665.s004.doc]

**Table S1. Expression of CXCR7 and CXCL12 in NB clinical groups.**

|  | **Neural cells** | | | | | **Endothelial cells** | | | | | **Stromal cells** | | | | |
| --- | --- | --- | --- | --- | --- | --- | --- | --- | --- | --- | --- | --- | --- | --- | --- |
| ***INSS stage*** | ***1*** | ***2*** | ***3*** | ***4*** | ***4S*** | ***1*** | ***2*** | ***3*** | ***4*** | ***4S*** | ***1*** | ***2*** | ***3*** | ***4*** | ***4S*** |
| ***CXCR7 expression*** | | | | | | | | | | | | | | | |
| Positive tumors (%) | 61 | 78 | 87 | 67 | 93 | 41 | 26 | 53 | 43 | 18 | 54 | 52 | 75 | 48 | 50 |
| Number of cases | 19 | 15 | 28 | 39 | 15 | 13 | 5 | 17 | 25 | 3 | 17 | 10 | 24 | 28 | 8 |
| Median score | 0.94 | 1.04 | 0.98 | 0.87 | 0.94 | 0.14 | 0.05 | 0.23 | 0.21 | 0.01 | 0.4 | 0.39 | 0.63 | 0.55 | 0.29 |
| *p-value* | *ns^(+)^* | | | | *ns^(-)^* | *ns ^(+)^* | | | | *0.004^(-)^* | *0.001*^(§)^ | *ns ^(+)^* | | | *0.0002^(-)^* |
| ***CXCL12 expression*** | | | | | | | | | | | | | | | |
| Positive tumors (%) | 77 | 89 | 93 | 84 | 87 | 100 | 100 | 100 | 100 | 100 | 100 | 100 | 100 | 96 | 93 |
| Number of cases | 24 | 17 | 29 | 49 | 14 | 31 | 19 | 32 | 58 | 16 | 31 | 19 | 32 | 56 | 15 |
| Median score | 0.77 | 0.89 | 0.84 | 0.79 | 0.98 | 3.09 | 3.05 | 3.16 | 3.16 | 3.06 | 1.95 | 2.45 | 1.97 | 1.93 | 1.91 |
| *p-value* | *ns^(+)^* | | | | *ns ^(-)^* | *ns ^(+)^* | | | | *ns ^(-)^* | *ns ^(+)^* | | | | *ns ^(-)^* |

INSS : International Neuroblastoma Staging System ; Median score means average tumor score, as established by semi-quantitative analysis of the immunostaining ; p-value (Student’s t-test) : ^(+)^ represents p-value between stages 1-2 and stages 3-4 ;  ^(-)^ represents p-value between stage 4 and stage 4s ; ^(§)^ represents p-value between stage 1 and stage 4 ; ns means not significant, p < 0.05 is considered significant, p ≤ 0.01 and p ≤ 0.001 are considered highly significant.
